# Beneficial Effect of Root or Foliar Silicon Applied to Cucumber Plants under Different Zinc Nutritional Statuses

**DOI:** 10.3390/plants10122602

**Published:** 2021-11-27

**Authors:** José María Lozano-González, Clara Valverde, Carlos David Hernández, Alexandra Martin-Esquinas, Lourdes Hernández-Apaolaza

**Affiliations:** Department of Agricultural Chemistry and Food Science, Universidad Autónoma de Madrid, Av. Francisco Tomás y Valiente 7, 28049 Madrid, Spain; josem.lozano@uam.es (J.M.L.-G.); clara.valverdes@estudiante.uam.es (C.V.); carlosd.hernandez@estudiante.uam.es (C.D.H.); alexandra.martin@uam.es (A.M.-E.)

**Keywords:** silicon, Zn-deficiency, Zn-sufficiency, Zn re-fertilization

## Abstract

Zinc (Zn) is an essential micronutrient involved in a large variety of physiological processes, and its deficiency causes mainly growth and development disturbances, as well as oxidative stress, which results in the overproduction and accumulation of reactive oxygen species (ROS). A possible environmentally friendly solution is the application of silicon (Si), an element that has shown beneficial effects under abiotic and biotic stresses on many crops. Si could be applied through the roots or leaves. The aim of this work is to study the effect of Si applied to the root or shoot in cucumber plants under different Zn statuses (sufficiency, deficiency, and re-fertilization). Cucumber plants were grown in hydroponics, with 1.5 mM Si applied at the nutrient solution or sprayed on the leaves. During the different Zn statuses, SPAD index, fresh weight, ROS, and Si, Zn, P, Cu and B mineral concentration were determined. The results suggested that Si application had no effect during sufficiency and deficiency periods, however, during re-fertilization foliar application of Si, it showed faster improvement in SPAD index, better increment of fresh weight, and a decrease in ROS quantity, probably due to a memory effect promoted by Si previous application during the growing period. In summary, Si application to cucumber plants could be used to prepare plants to cope with a future stress situation, such as Zn deficiency, due to its prompt recovery after overcoming the stress period.

## 1. Introduction

Monosilicic acid (H_4_SiO_4_) is the plant-assimilable form of silicon (Si), which can be found in soils with a concentration ranging between 0.1 and 1.4 mM [1]. Although all soil-grown plants contain some Si in their tissues [2], plants have different capacities to accumulate it, with values varying between 0.1% and 10% Si (dry weight) [3]. This element has been classified as a beneficial but non-essential nutrient for higher plants [1,2,4,5], as some species are almost unaffected by silicon fertilisation compared to others [6], and with different expression and functionality of Si transporters [7]. However, the beneficial effect of Si on the plant growth promotion under biotic and abiotic conditions has been extensively studied [2,8,9,10,11,12] in both root [13,14,15] and shoot [16,17,18] application. As discussed above, many studies have been conducted on the beneficial effects of Si in plants, but the mechanism of action of this element is still under discussion. Coskun et al. [6] proposed that the effect of Si is due to Si deposits formed in the apoplast, triggering the activation of the plant’s stress responses. Hernández-Apaolaza et al. [17] also noted that the application of Si appears to be related to the induction of the corresponding stress responses, at least under micronutrient deficiencies, and included the hypothesis of the activation of some kind of memory effect that was activated by the Si addition, which was evident under resupply experiments. In any case, further studies are needed to clarify and prove these emerging theories.

Zinc (Zn) is one of the essential microelements for plants, which has a functional and structural role in enzymatic reaction, being part of numerous enzymes, such as: carbonic anhydrase, responsible for CO_2_ fixation in photosynthesis; alcohol dehydrogenase, that converts acetaldehyde to ethanol in anaerobic respiration in roots; or superoxide dismutase (SOD), which protects the plant against oxidation by superoxide radicals [19]. It also is involved in the synthesis of tryptophan, a precursor of the hormone auxin (IAA) necessary for plant growth [20], has an essential role in maintaining the structure and permeability of the plasma membrane [21], and is involved in the transport of phosphorus through the plant [19]. The high pH in calcareous soils decreases the solubility of the metal, thus precipitating it in the form of carbonates or hydroxides [22], decreasing its bioavailability, and causing a deficiency of this element in plants. Zn deficiency generates a delay in the growth of the plant, decrease and malformations in the young leaves, shortening of the internodal distance and, in the case of a severe deficiency, chlorosis and necrotic spots [1,23]. Furthermore, Zn deficiency can give rise to oxidative stress, which triggers an over-production and accumulation of reactive oxygen species (ROS), species that, in excess, can behave as toxic compounds [24]. Additionally, Zn deficiency is related to a higher accumulation of phosphorus in the old leaves; the more severe the Zn deficiency, the higher the phosphorus concentration [25]. There are different strategies to alleviate Zn deficiency, such as the application of inorganic salts to the soil or synthetic chelates among others [26], however, these strategies are expensive and have a high environmental impact. Another environmentally friendly alternative to alleviate Zn deficiency symptoms could be Si fertilization.

Bityutskii et al. [13] tested the effect of root Si addition on Zn-deficient cucumber (*Cucumis sativus* L.); for this, plants were grown for 2 weeks in hydroponics with or without Zn, Si was added as 1.5 mM H_4_SiO_4_. They observed that Zn deficiency symptoms were only partially prevented because no necrotic spots were observed, which may be relayed to an enhanced antioxidant capacity. Mehrabanjoubani et al. [27] observed that the application of Si in the form of sodium metasilicate (Na_2_SiO_3_·5H_2_O) increased: nutrient uptake, shoot biomass, and grain yield in rice plants submitted to Zn deficiency, toxicity and optimal Zn levels. It is commonly known that Si is essential for rice growth. Moreover, silicon application increased Zn, as well as Ca^2+^, K^+^, P and B contents in plants supplied up to 50 µg L^−1^ Zn. In the experiment conducted by Pascual et al. [28] 0.5 or 1.0 of Na_2_SiO_3_·5H_2_O was added to the nutrient solution of Zn-deficiency soybean (*Glycine max* L.) plants; after each sampling, Zn concentration in root, stem and leaves were measured. It was observed that treatment with 0.5 mM of Si promoted Zn accumulation in the root apoplast, and its subsequent remobilization by shoot, ameliorating the Zn deficiency symptoms. These authors developed a preculture with Si addition before the Zn depletion from the nutrient solution.

The aim of this work is to evaluate, for the first time, as far as we know, the effect of foliar and root application of Si in cucumber plants under different Zn statuses: Zn sufficiency, Zn deficiency, and Zn re-fertilization. Moreover, the effect of Si in other nutrient content (Cu, P, B) was also studied under the three Zn statuses. P concentration was studied for the well-known antagonism between this element and Zn, and Cu was evaluated due to its presence in the Cu/Zn SOD enzyme, which among others is uncharged, to decrease ROS concentration generated by Zn deficiency (and the rest of biotic and abiotic stressors), and finally, B has been studied due to its chemical similarities to Si.

## 2. Results

Silicon addition to the root (+SiR) or shoot (+SiF) of cucumber plants has been evaluated under Zn sufficiency (+Zn), deficiency (−Zn), and re-fertilization (−Zn(+Zn)). Two samplings were carried out: sampling 1 at the end of the Zn deficiency period, and sampling 2 at the end of the re-fertilization period. In each sampling, plants were compared with their respective controls (−Si+Zn and −Si+Zn(+Zn)).

### 2.1. Effect of Silicon on SPAD Index and Biomass under Different Zn Nutrient Statuses

**Figure 1:**
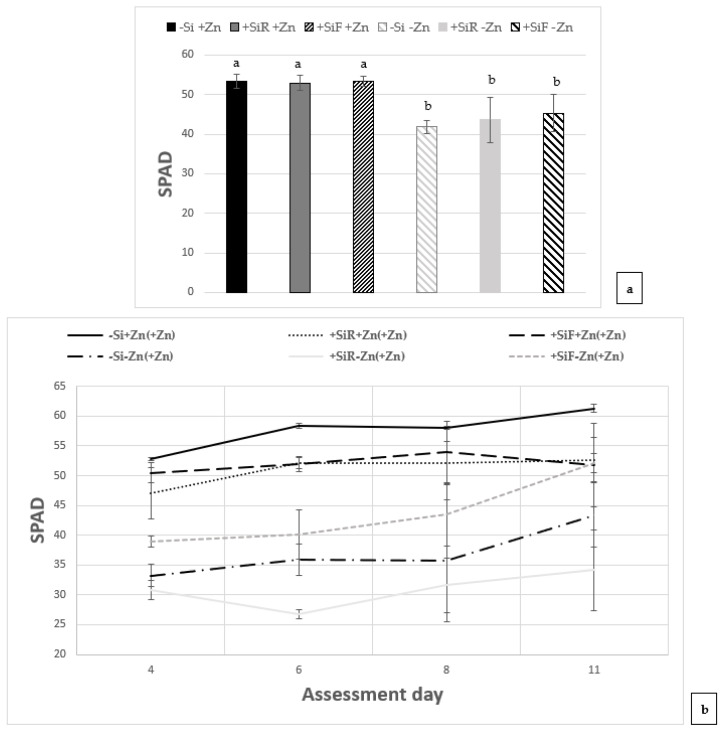
Effect of Si addition on the root (+SiR) or on the shoot (+SiF) of cucumber plants grown under different Zn nutritional statuses on SPAD values: (**a**) After a Zn deficiency period (−Zn) compared to the control plants with continuous Zn addition (+Zn) and (**b**) During a Zn re-fertilization period (−Zn(+Zn)) compared with their corresponding control plants (+Zn(+Zn)). The data are the mean ± SE (*n* = 5). Different letters indicate significant differences according to Duncan’s test (*p* < 0.05).

The SPAD index was assessed in new leaves after the Zn deficiency period (sampling 1, Figure 1a), and at different days (4, 6, 8 and 11) during the Zn resupply period (Figure 1b), and compared to their respective controls (+Zn for sampling 1 and +Zn(+Zn) for sampling 2). In sampling 1, +Zn treatments showed significantly higher SPAD index values than the −Zn treatments. Among the treatments with +Zn, no differences were observed with or without Si application, and the same occurred in the deficient treatments (−Zn). In sampling 2 (Zn resupply), treatments with a continuous Zn addition (+Zn(+Zn)) showed significantly higher SPAD index than plants that have suffered a previous period of Zn deficiency (−Zn(+Zn)), being the treatment without Si application (−Si+Zn(+Zn)), the one with the highest SPAD index. The application of Si to the leaves after a period of Zn deficiency followed by a Zn resupply (+SiF−Zn(+Zn)) showed significantly better results than the other Si treatments (−Si−Zn(+Zn) or +SiR−Zn(+Zn)), presenting similar levels to the treatments with a continuous Zn application (+SiR+Zn(+Zn) and +SiF+Zn(+Zn)), at day 8 and 11 after Zn resupply (Figure 1b).

With respect to plant biomass (Table 1), plants were divided into root, stem, old leaves (grown before the Zn depletion from the nutrient solution in sampling 1 or grown before the refertilization in sampling 2), and new leaves (grown during Zn deficiency and resupply periods). In sampling 1 (after Zn deficiency), control plants (+Zn) showed significantly higher fresh weight (FW) values than the −Zn ones. In both sufficiency (+Zn) and deficiency (−Zn) statuses, the application of Si (+SiR and +SiF) had no effect; also, in both cases, it was observed that the highest percentage of fresh weight was located in the root. After Zn resupply (sampling 2), control treatments with +Zn(+Zn) have a significantly higher fresh weight than treatments that have undergone a period of deficiency (−Zn(+Zn)). In the treatment (+Zn(+Zn)), the application of Si (+SiR and +SiF) significantly increases fresh weight compared to the treatment without Si (−Si), the foliar application (+SiF) being the treatment that presented the highest fresh weight values. With respect to the −Zn(+Zn) treatments, the application of Si (+SiR and +SiF) had no effect. As in sampling 1, most of the fresh weight was located in the root (Table 1). In addition, in the treatments with Zn-refertilization (−Zn(+Zn)), an important percentage of the fresh weight was also located in the new leaves (Table 1).

### 2.2. Effect of Silicon on ROS under Different Zn Nutrient Statuses

**Figure 2:**
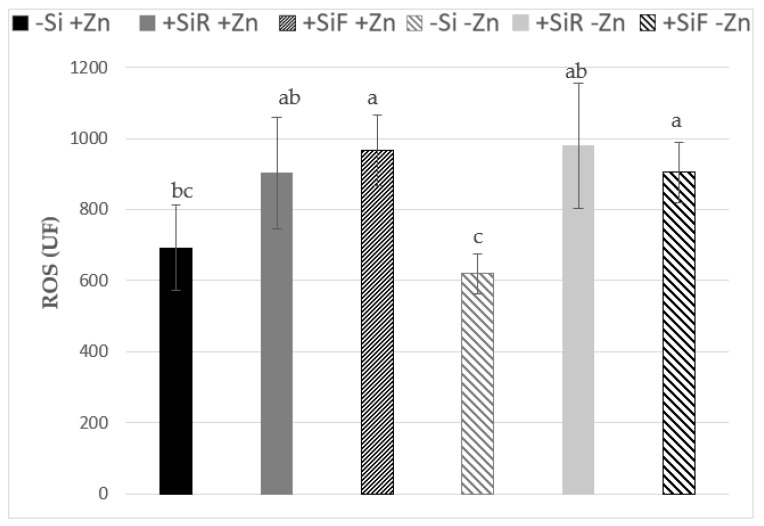
Effect of Si addition on the root (+SiR) or on the shoot (+SiF) of cucumber plants grown under different Zn nutritional statuses on reactive oxygen species (ROS) (FU). Plants were collected after a Zn deficiency period (−Zn) and compared to the control plants grown with continuous Zn addition (+Zn) after 16 days of growth. The data are the mean ± SE (*n* = 9). Different letters indicate significant differences according to Duncan’s test (*p* < 0.05).

Reactive oxygen species (ROS) are an indication of oxidative stress in a plant; the more ROS there are, the greater the oxidative stress. A decrease in ROS indicates the activation of the plant’s antioxidant defense capacity. After Zn deficiency, control plants treated with foliar Si (+SiF+Zn) seemed to be more stressed than control plants without Si application (−Si+Zn) (Figure 2), but when Si was applied to the root, no differences were observed. However, in Zn deficient plants (−Zn), Si addition, either to roots or leaves, increased ROS concentration in these tissues.

To study the effect of Si addition on Zn resupply response, the variation of ROS was calculated between both statuses (Zn deficiency and resupply) by subtracting the ROS after the Zn resupply period (Sampling 2) from the ROS, after the Zn deficiency period (Sampling 1). Moreover, this ROS increment was compared with the ROS variation in control plants which did not suffer a Zn deficiency period (+Zn(+Zn)) (Table 2). Both types of Si application reflected a significant decrease in ROS increment, being the Si foliar addition, the one which reduces ROS after Zn resupply in a greater amount. These results indicated that, after overcoming a period of Zn deficiency, the foliar application of Si (+SiF) helped the plant to decrease its ROS concentration.

### 2.3. Effect of Silicon on Mineral Concentration in Plant Tissues under Different Zn Nutrient Statuses

The concentrations of silicon (Si), zinc (Zn), copper (Cu), boron (B) and phosphorus (P) in plant tissues (root, old leaves and new leaves) were determined at the end of the Zn deficiency period (sampling 1) and Zn re-fertilization period (sampling 2).

**Figure 3:**
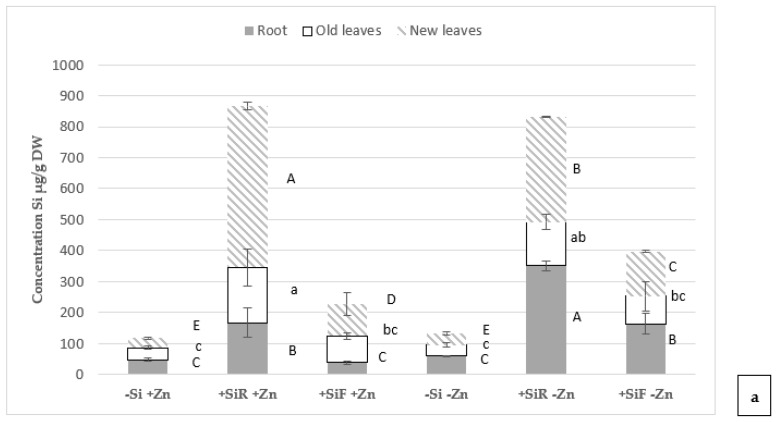

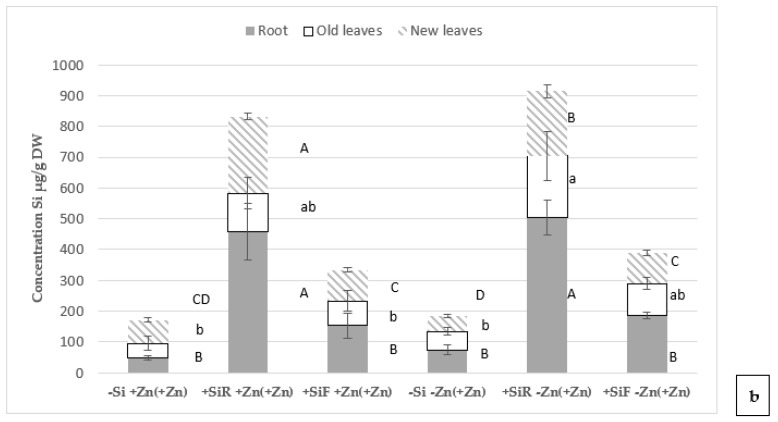
Effect of Si addition on the root (+SiR) or on the shoot (+SiF) on the Si concentration (µg g^−1^ DW) in root, old and new leaves of cucumber plants (**a**) After a Zn deficiency period (−Zn) compared to the control plants with a continuous Zn addition (+Zn) and (**b**) After a Zn re-fertilization period (−Zn(+Zn)) compared with their corresponding control plants (+Zn(+Zn)). Data are mean ± SE (*n* = 9). Different letters indicate significant differences for each plant organ according to Duncan’s test (*p* < 0.05).

Regarding the Si concentration at the end of the Zn deficiency period (Figure 3a), the −Si+Zn treatment showed the lowest concentrations, and the treatment with root application of Si (+SiR+Zn) showed higher concentrations in all plant organs than the foliar application (+SiF+Zn). The same trend could be observed in the treatments where Zn deficiency was generated (−Zn). The results obtained indicated that more Si was absorbed in Zn-deficient plants, although it was more poorly remobilized in the plant, since the concentration of Si in the root after root or foliar application of Si (+SiR and +SiF) in the Zn-deficient treatments (−Zn) was higher than in the counterpart treatments with sufficient Zn (+Zn); however, the opposite occurred with respect to the concentration of Si in the aerial part. On the other hand, these results indicated that Si was better absorbed and remobilized when applied by root (+SiR) than when applied foliar (+SiF). After Zn re-fertilization (sampling 2) (Figure 3b), the trend in Si concentration in the different plant tissues was similar to that observed after the deficiency period (sampling 1), Si concentration in the treatment without Si application (−Si) was the lowest, Si concentration in the treatments where it was applied to the root (+SiR) was higher in all tissues than in the treatments where it was applied to the leaves (+SiF); this trend was observed both in plants with a continuous supply of Zn (+Zn(+Zn)), and in those with a period of deficiency (−Zn(+Zn)).

In relation to the total Zn concentration in the whole plant at the end of the Zn deficiency period (sampling 1) (Table 3), in the control treatments with continuous Zn supply, no differences due to Si application have been found. However, Zn distribution was affected by the type of Si application. Compared to the control treatment (−Si+Zn), root Si addition (+SiR+Zn) decreased Zn percentage in root, but no differences were obtained in old or new leaves, although with foliar Si supply (+SiF+Zn), Zn significantly increased in new leaves. In sampling 2, plants with foliar Si and continuous Zn supply (+SiF+Zn(+Zn)) significantly increased the total Zn concentration with respect to −Si plants, although no differences with Si applied to the roots were obtained. According to Zn distribution in plant organs, Si enhanced Zn accumulation in root with both application types. Under Zn deficiency (sampling 1), only foliar Si application maintained similar Zn levels than in sufficiency (Table 3), and this Zn was mainly accumulated in new leaves. In the Zn resupply period (sampling 2), both Si treatments (+SiR−Zn(+Zn) and +SiF−Zn(+Zn)) enhanced Zn restoration to sufficiency levels compared with the control plants (−Si+Zn(+Zn)), and no statistical differences were observed between them. Interestingly, Zn was mainly in new leaves in +SiF treatment and in roots in +SiR treatment after re-fertilization. Related to P concentration, in sampling, 1 Zn depletion from the nutrient solution did not affect P concentration in the whole −Zn plants (Table 3). Phosphorous percentage in new leaves decreased in −Zn conditions without Si addition to the media but increased when Si was applied to the leaves. In sampling 2, after plant recovery, no differences in total P concentration in the entire plant were obtained in −Zn(+Zn). In root, +SiR−Zn(+Zn) treatment had the highest percentage of P; meanwhile, in the +SiF−Zn(+Zn) one, P was accumulated in new leaves (Table 3). In +Zn conditions at both samplings, data for roots showed that +SiR addition enhanced P percentage compared with the control plants (−Si+Zn and −Si+Zn(+Zn)); in old leaves, plants without Si drastically increased P percentage; although when Si was applied, this increment was not observed.

Copper (Cu) and boron (B) concentrations in the entire plant at the end of Zn sufficiency, Zn deficiency and Zn re-fertilization periods in all treatments were measured (Table 4). With respect to the total Cu concentration in the whole plant at sampling 1, among the +Si treatments of Zn sufficient plants, the +SiR+Zn one showed the highest concentration of Cu, and it was mainly located in new leaves. In sampling 2, the treatment with foliar application of Si (+SiF+Zn(+Zn)) presented the lowest concentration of Cu in the whole plant. In this sampling, the distribution of Cu in the Si treatments was mainly in the root, while in the control plants (−Si+Zn(+Zn)), Cu was found in the new leaves. The concentration of Cu in the treatments under Zn deficiency (sampling 1) was higher than in the Zn sufficiency plants. Under Zn deficiency, the treatment with root application of Si (+SiR−Zn) showed the lowest concentration. Plants without Si application (−Si−Zn) and with root application of Si (+SiR−Zn) presented a higher percentage of Cu in new leaves; however, after foliar application of Si (+SiF−Zn), Cu was found mainly in the root. In the Zn resupply period (sampling 2), Cu concentration attained much higher values than the control treatment (−Si+Zn(+Zn)), except for the root application of Si (+SiR−Zn(+Zn)), which showed similar values to the control. In all treatments, Cu was mainly located in the root. In relation to B concentration, in sampling 1 treatments, with or without Si application and with an optimal or deficient Zn nutritional level (+SiR+Zn; +SiF+Zn), they did not present significant differences with respect to control plants in the concentration of B in the entire plant (Table 4). Regarding the distribution of B in the plant, in all treatments B, was found mainly in the old leaves. In sampling 2, Si addition significantly increased B concentration in the plant compared to untreated plants in well fed plants. Under Zn resupply, +SiF−Zn(+Zn) presented the highest B concentration in the plant, and this element was found mainly in old leaves.

## 3. Discussion

Different effects could be observed in the parameters measured during the study, depending on the nutritional status of the cucumber plant and the treatment applied, so discussion has been divided into Si impact on Zn sufficient, deficient, and re-fertilized plant for each determination.

### 3.1. Effect of Silicon on SPAD Index and Biomass under Different Zn Nutrient Statuses

For the first time, the effects of Si applied to the root (+SiR) and leaves (+SiF) were tested on cucumber plants that underwent periods of Zn sufficiency, deficiency, and Zn deficiency recovery. In the plants that had a continuous supply of Zn in sampling 1 (16 days of growth), no differences have been observed in the SPAD index, although in sampling 2 (27 days of growth), the application of Si to both root (+SiR+Zn(+Zn)) and leaves (+SiF+Zn(+Zn)) provided a significantly lower SPAD index compared to the control (−Si+Zn(+Zn)) (Figure 1). Hernández-Apaolaza et al. [17], in a similar experiment in which cucumber plants were grown with Si applied both foliar and to the roots to well nourished seedlings, only found a decrease in the SPAD index with respect to the control plants when Si was applied to the roots (+SiR+Fe). These authors grew the plants under optimal nutrient conditions for 10 days, but in the present work, only 7 days of growth with a completed nutrient solution were applied. This fact may explain why the treatment with foliar Si did not suffer an SPAD decrease at that point. Several authors have argued that Si application enhances the Casparian Band of the root exodermis development [6,29,30,31,32]; this increase of apoplastic barriers may hinder nutrient uptake such as iron [33,34,35], and maybe Zn, which could explain the low SPAD levels compared to the Si-untreated plants, as it is well known that Zn deficiency promotes chlorosis in leaves [1]. This parameter may indicate that Si, when applied to the root, promoted Zn deficiency even when plants were submitted to a complete nutrient solution, as it was described before by Hernandez-Apaolaza et al. [17]. However, results obtained at this stage (Sampling 2) showed that plants with foliar Si (+SiF+Zn(+Zn)) significantly increased the total Zn concentration in the plant (Table 3) with respect to −Si plants, although no differences when Si was applied to the roots were obtained. However, the Zn distribution in plant organs showed that Si enhanced Zn accumulation in root with both application types, so this may explain the lower SPAD index found when Si was added. SPAD results of Zinc deficient plants (Figure 1a) indicated that Si addition did not have any influence on this parameter under −Zn. However, when Zn concentration is taken into account (Table 3), plants with foliar Si presented a significant higher amount of Zn than the other Si treatments, and were similar to control plants without Si and +Zn. In this case, Zn was equally distributed between roots and new leaves (Table 3). Likewise, Farias Guedes et al. [36] observed a significant increase in chlorophyll content, with foliar applications of Si to Zn-deficient Sorghum plants. Furthermore, the studies of Pascual et al. [28] and Bityutskii et al. [13] with soybean and cucumber plants, respectively, did not show significant increases in SPAD or chlorophyll content when Si was added to the nutrient solution (+SiR).

In plants that underwent a Zn deficiency period followed by a recovery one (−Zn(+Zn)), the treatment with foliar application of Si showed significantly higher SPAD levels than the other Si treatments (Figure 1), similar to that observed by Hernández-Apaolaza et al. [17] with Fe resupply of cucumber plants. These authors hypothesized that this quicker recovery from the chlorosis, was possibly related to plant memory effect. Srivastava et al. [37] observed that plants have to cope with different stresses all along their crop cycle, and retained ‘memories’ of previously encountered stresses as an adaptive mechanism that help them to confront forthcoming stresses more rapidly and efficiently. Such memories can be induced artificially, through preexposure to low-dose stressor, or by the addition of beneficial compounds like silicon. The induced stress memory is called ‘acquired tolerance’, and it can be retained either in the short term (somatic memory), or may be transferred to succeeding generations (intergenerational memory), or in some cases, inherited across generations (trans-generational memory) [37]. Ding et al. [38] applying successive dehydration stress/recovery treatments in a relatively short time in Arabidopsis plants, pointed out that leaf cells during recurring dehydration stresses displayed a transcriptional stress memory. During recovery (watered) states, trainable genes produce transcripts at basal (preinduced stress) levels, as expected, but remain associated with atypically high stress marks, that reinforced the response to the rewatering process. Our results may conduct a similar plant response related to Zn resupply. With respect to Zn concentration (Table 3), both +SiR−Zn(+Zn) and +SiF−Zn(+Zn) presented a tendency of Zn accumulation in the plant compared to −Si−Zn(+Zn), although only the +SiR treatment had a significant higher amount of Zn; even though Zn distribution in plant organs pointed to new leaves of +SiF having the greatest Zn percentage, which would explain this treatment having the highest SPAD index.

With respect to biomass (Table 1), in sampling 1, no significant differences were found in plants that had a correct Zn nutritional status (16 days +Zn); nevertheless, after 27 days of growth (sampling 2) the application of Si to the roots or shoots significantly increased the fresh weight compared to the treatment without Si application, +SiF being the treatment that produced the greatest increase. These results pointed to the beneficial effect of Si on biomass increase, especially if Si was applied to the leaves. A period of more than 16 days of growth was required to observe the beneficial effect of Si on biomass. Similar results were obtained by Mehrabanjoubani et al. [27] in rice plants, when different Zn concentrations (1, 10, 50 and 100 µg L^−1^) were applied, and root Si was added as sodium silicate (1.5 mM) in hydroponics. They observed a significant increase in biomass in plants, with an optimal Zn nutritional status due to the application of Si after 105 days of treatment. Zn is an essential micronutrient necessary for optimal growth [1], and as expected, the observed increase in biomass was well correlated with significant increase in Zn concentration in the whole plant in +SiF+Zn(+Zn) plants, with respect to the control plants (−Si+Zn(+Zn)) (Table 3). As mentioned before, the apoplastic obstruction caused by Si and described by Coskun [6] was avoided when Si was applied to the leaves. This fact may explain why these plants presented the highest fresh weight values. On the contrary, Si application had no effect on fresh weight under Zn deficiency conditions (Table 1), which were consistent with the results obtained by Pascual et al. [28] in soybean plants. However, Mehrabanioubani et al. [39] observed that root Si addition increased total weight in −Zn rice plants. These differences among experiments were probably due to the different plant species used, and their ability to accumulate Si. Plants with active uptake of Si are classified as high accumulators, with passive silicon uptake are classified as intermediate accumulators, and with rejection mechanisms, are classified as non-accumulators [40]. Rice is known to be a high Si accumulator [41], and both soybean and cucumber are intermediate accumulators [42,43]. The total Zn concentration in the +SiF−Zn plants (Table 3) had a significantly higher concentration of Zn than the other treatments (−Si−Zn and +SiR−Zn), though a higher fresh weight was not recorded. In plants to which Zn was added back into the nutrient solution (−Zn(+Zn)), no significant differences in fresh weight were observed (Table 1), but fresh weight and Zn concentration (Table 3) values tend to be higher in the treatments with Si application. Maybe, if the period of re-fertilization were extended, significant differences in fresh weight could be observed.

### 3.2. Effect of Silicon on ROS under Different Zn Nutritional Statuses

Reactive oxygen species (ROS) are regulated naturally by the action of enzymes such as catalase and superoxide dismutase [44], or by non-enzymatic mechanisms, such as the accumulation of phenols [45,46]. Several authors have reported the improvement of the antioxidant defense capacity of the plant due to the application of Si to the root, as enzymatic activities such as SOD and CAT have been improved by the application of Si, which would cause a ROS reduction [47,48]. In addition, as with root application (+SiR), foliar application (+SiF) also stimulates the activation of antioxidant defenses [49,50,51]. Here, plants with continuous Zn supply and treated with foliar Si were more stressed than the −Si plants after 16 days of growth (sampling 1; Figure 2), but the Zn concentration in the whole plant was similar in all the treatments (Table 3). These data have been supported by Hacisalihoglu et al. [52], who observed no differences in shoot Zn concentrations between the Zn-inefficient and Zn-efficient bean genotypes grown under low−Zn conditions, where differences in Zn efficiency were exhibited. Zn efficiency is defined as the ability of plants to maintain high yield under Zn-deficiency stress. However, at the end of the experiment (27 days) no significant differences between treatments were found in ROS variation values, which decreased for all of them when Zn was resupplied to the nutrient solution (Table 2). Even though there were no stress situations, plants with Si application presented higher ROS (specially with Si foliar application) at the first sampling time. After that period, plants will be recovered (Table 2) and probably show the beneficial effect of this element at longer testing periods. Different results were obtained by de Farias Guedes et al. [36] in sorghum, where they observed that plants with a correct nutritional status sprayed with Si decreased their cell damage. In their experiment, the effect of Si on oxidative stress was studied 111 days after emergence, so the difference in the experiment duration, as well as the plant species used, may explain the disparity of the results. ROS levels in Zn-deficient plants with both Si applications (+SiR−Zn; +SiF−Zn) were significantly higher than in Si untreated plants (−Si−Zn) (Figure 2). It is well known that Zn deficiency produces an increment of ROS [20], and due to the results obtained, both types of Si application increased plant stress, since they presented significantly higher levels of ROS compared to the treatment without Si application (−Si). The results obtained for Zn sufficiency at first sampling time, and at Zn deficiency, were in agreement with the previously explained hypothesis of apoplastic obstruction. On the other hand, if after a period of Zn deficiency, Zn was added back to the nutrient solution, the treatments with Si application showed an improvement in the mitigation of ROS concentration in new leaves; specifically, the effect was more relevant in +SiF plants (Table 2). The data obtained suggest that there was a significant improvement in plant antioxidant defense capacity with foliar Si application, which was attributed to the “memory effect” of the plants [38], that “remember” the previous stress caused by the Si addition and were better prepared to cope with it than the non−Si-treated plants.

Silicon application improves the accumulation of Cu-ligands, such as proteins like Zn/CuSOD [53], which will contribute to ROS concentration reduction. There are three types of superoxide dismutase enzymes that are described in plants: FeSOD, MnSOD, and Cu/ZnSOD (where Cu is the redox active catalytic metal). However, the overproduction of superoxide dismutase only gives limited protection to abiotic stress, and does not remarkably improve plant performance [54]. Surprisingly, plant lines lack the most abundant Cu/ZnSOD or FeSOD activities performed, as well as the wild-type under most conditions tested, indicating that these superoxide dismutases were not limiting to the prevention of oxidative damage. As Cu was the catalytic metal involved in SOD activity, Cu was measured either for sufficient, deficient, or refertilized plants. In general, under Zn sufficiency, foliar Si decreased Cu concentration in plant (Table 4), and no Cu accumulation in roots was observed, as described by Bosnić et al. [53]. The expected increase in ROS concentration in the +SiF+Zn due to Cu decrease was obtained after 16 days of plant growth (Figure 2), although this has not reflected at the end of sampling 2 (Table 2), which supported the Pilon et al. [54] statement that SOD was not really a limiting factor to prevent oxidative damage. Comparing these data according to Zn concentration results (Table 3), in sampling 1 no differences in Zn concentration was observed, so the copper effect was the main fact that controlled Cu/Zn SOD activity. In sampling 2, +SiF significantly increased Zn concentration in the plant compared to −Si+Zn plants, so this may compensate for the decreased in Cu concentration found for this treatment, and finally show similar behavior according to ROS accumulation. Zinc-deficient plants showed that +SiR−Zn had the lowest Cu concentration in plant; subsequently, a high ROS concentration was found, although no statistical differences with +SiF−Zn were obtained. Moreover, the +SiF treatment was the one that accumulated the most Cu in the root. This may be due to the fact that foliar-applied Si enhances Cu binding capacity to the cell wall, and the accumulation of Cu-ligands, more than root-applied Si [55]. As mentioned above, Pilon et al. [54] indicated that the lack of the most abundant Cu/ZnSOD or FeSOD activities is not limiting, but a strong defect in chloroplast gene expression and development appeared in plants that lack the two minor FeSOD isoforms, which are expressed predominantly in seedlings, and that associate closely with the chloroplast genome. On the contrary, several authors have reported that Fe deficiency enhanced SOD activity [8,56,57], but the literature did not distinguish between the FeSOD isoforms altered. In refertilization, −Si plants presented the highest ROS increment, which means that ROS concentration produced due to −Zn was still present in the plants. Their Cu concentration was the highest among treatments, but its Zn concentration was the lowest, along with foliar Si treatment. This shows that the Zn concentration plays an important role in ROS scavenging, related to Cu/ZnSOD activity. As can be seen in the distribution of Cu in plant tissues (Table 4), the +SiF treatment had most of the Cu lodged in the root, which could provide evidence for an improvement in the Cu binding capacity of the root cell wall; this may have enhanced Zn/CuSOD accumulation, which would help to decrease oxidative stress. To verify this fact, future experiments could measure the amount of SOD isoforms present in the plant in such conditions.

### 3.3. Effect of Silicon on Mineral Concentration in Plant Tissues under Different Zn Nutritional Statuses

Concentrations of Si at the different Zn statuses were measured (Figure 3), and the first relevant thing observed was that, when Si was applied through the roots, a significantly higher amount of Si was measured than when applied to the shoot. It was necessary to note here that foliar Si addition tested was at the optimal levels reported in previous works (for example [12,17,58]) for different crops. Therefore, the Si amount added through root and leaf applications was not the same. When Si was added to the nutrient solution, the concentration was 1.5 mM, and it was renewed weekly, however, when Si was added to the leaves by spraying, three doses (125 µL each) per leaf per week were used. The difference was due to the instability of the Si solutions at the high concentrations required to spray shoots, if a similar amount of Si should be added through roots and shoots. When Si concentration in Zn-sufficient and -deficient plants were compared (sampling 1) (Figure 3a), a significantly higher accumulation of Si in the root at Zn deficiency treatments was observed. In Figure 3b, resupply and sufficient plants only differ in the Si root treatment, in which Si in new leaves were lower in the resupply experiment than in the sufficient one.

Many attempts have been made to reveal the mechanisms of Zn-efficient plants in response to low−Zn, but they are still not well described [59]. As Hacisalihoglu [59] described in his review, several uptake Zn^+2^ studies in crop plants found no strong correlation between root influx and Zn efficiency, which indicates that Zn efficiency in higher plants is likely not a root-focused trait, but a shoot-focused trait, possibly related to shoot Zn compartmentation; because efficient plants maintain higher cytoplasmic Zn concentrations under Zn deficiency conditions, which provided enough elements to continue with the numerous cytoplasmatic localized physiological processes that require Zn [52]. As observed by Bityutskii et al. [13] in cucumber plants, root application of Si in Zn deficient plants had no effect on Zn concentration and mobility in plant tissues, which is confirmed in this study. In plants to which Zn was added back to the nutrient solution (−Zn(+Zn)), the concentration of Zn in the treatment with root application of Si (+SiR) showed significantly higher values than the treatment without Si application (−Si). No significant differences were observed between the Si treatments. These results suggested that root application of Si after a period of Zn deficiency, significantly increases the concentration of this element compared to the non-application of Si. Similar findings were observed by Hernández-Apaolaza et al. [17] in a Fe resupply assay.

It has been found that Si improves P uptake by the root, and a subsequent increase in soluble inorganic P concentration in leaves of wheat and corn, and the enhancement of the utilization of P within the plant tissues under P deficiency conditions was described [60,61]. In +Zn plants in sampling 2, root application of Si (+SiR) showed significantly higher concentration of P (Table 3) than the other treatments. However, +SiR treatment presented the highest fraction of P in the root, indicating that Si improved P uptake; although, no remobilization to the leaves was observed. Silicon stimulated root Pi acquisition by increasing the exudation of carboxylates [60], although the effect of exuded carboxylates on P-mobilization in the soil is still controversial [62]. Neither in deficiency nor re-fertilization periods were significant differences in P concentration found; although under Zn deficiency, an overaccumulation of P in the aerial part was expected according to the literature [63]. It is interesting to note that, at −Zn treatments, a higher accumulation in the shoot of the plant was detected, similar to what was described by [60]. Moreover, in Zn deficiency and resupply periods, the −Si and +SiR treatments had more P fraction in old leaves, and the +SiF treatment in new leaves. This may be due to the effect of foliar application of Si on the mobility of P to the new leaves.

Keller et al. [64] found that Si induces Cu accumulation in the root epidermal cells, thus limiting root-to-shoot Cu translocation in wheat (Triticum turgidum) seedlings. The author proposed an increase in the Cu adsorption onto the root surface and immobilization in the vicinity of root epidermis, and a limitation of translocation through the thickened Si-loaded endodermis areas. Moreover, Bosnić et al. [53] provided evidence that the binding of Cu to the Cu-chelating proteins, such as Zn/Cu SOD in roots and plastocyanin in leaves, are important components of the Si-alleviating mechanism in cucumber exposed to Cu excess. With respect to +Zn treatments, in sampling 1, Cu concentration (Table 4) was significantly higher when Si was applied to the root (+SiR), but this effect disappeared in sampling 2. However, a significantly higher fraction of Cu in the root was observed in the Si-applied treatments (sampling 2), which may be due to the improved binding capacity of Cu to the root cell wall previously described [53,55,64]. In Zn-deficient plants, no accumulation of Cu was observed, due to Si addition (Table 4). Cu concentration, in plants with Zn re-fertilization, was significantly higher in treatment without Si. Furthermore, the Cu concentration in +SiF treatment, it was significantly higher than in +SiR treatment. In the deficiency period, in the re-fertilization, the treatment with foliar application of Si (+SiF) also presented the highest Cu fraction in the root.

Several authors [65,66] proposed the existence of a certain degree of competition within the B transport system, favoring Si uptake, due to both elements showing considerable chemical similarities. The effect of Si in decreasing B accumulation has been reported in numerous species [67,68,69]. A possible mechanism could be the formation of Si complexes with B in soil/nutrient solution, thus reducing the availability of B [70]. On the other hand, Rogalla and Römheld [71] reported that the application of Si to cucumber exposed to high B had no effect on total B concentration. However, Loomis and Durst [72] supported that there were positive correlations between Si and B uptake within different barley genotypes. Thus, genotypes with lower Si uptake also showed lower B uptake. At the three different Zn statuses, Si foliar application significantly increased B concentration in the whole plant (Table 4). Although the effect of +SiR was not clear, insufficient B availability affects several physiological and metabolic processes in plants, such as cell wall and plasma membrane structure and function, phenolics and nitrogen metabolisms, secondary metabolism and oxidative stress, gene expression, shoot and root growth (see [73]). Therefore, Si application leads to higher B concentration in the whole plant, and these will cause the cell wall and plasma membrane to become more structured, and then more prepared to cope with different stresses (in this case, Zn deficiency). Moreover, as B controls the phenolic compounds biosynthesis, and these compounds are related to antioxidant activity (ROS decrease), a relationship between Si, ROS, and B could explain the Si effect on ROS scavenging in refertilization. Further research about the interaction between Si, ROS and B for different plant species and nutrition statuses is needed.

To sum up, Zn sufficiency plants, as expected, presented the highest SPAD index values at both sampling times, but Si addition significantly reduced this index. However, this fact was not related to the Zn concentration in the whole plant, as Zn concentration in the +SiF plants was significantly higher with respect to the −Si control plants. This Zn concentration was mainly located at the roots; when Si was added either to the root or to the shoot, which could explain the lowest SPAD index found when Si was applied. Si supply increased fresh weight; especially the foliar application. Silicon addition to plants grown under optimal nutritional conditions, did not present clear advantages or disadvantages according to ROS values as stress indicators; however, Si influenced P, Cu, and B concentrations. Then, analyzing the Zn-deficient plants results, it can be seen that the Si addition did not improve the SPAD index, the fresh weight, or the P concentration. Moreover, the plants treated with Si were more stressed than the control without this element, although the Zn and B concentration in +SiF−Zn showed levels like Zn sufficient plants. This fact did not support the apoplastic obstruction theory given for Fe, that explained the lower uptake of this element when Si was added to the nutrient solution. In conclusion, Si addition to Zn-deficient plants did not show clear benefits on ameliorating its symptoms. Finally, a distinctive benefit of Si addition has been observed on refertilized plants, as foliar Si supply improves SPAD index, drastically reduced ROS concentration, and increased Zn and B concentration in plants, to levels similar to those of plants which did not suffer from Zn deficiency. This effect was slightly lower when Si was applied through the root, and could probably be due to the “memory effect” of the plants. These results suggest that foliar Si application to cucumber plants could be used to prepare plants to cope with a future stress situation, such as Zn deficiency, and to give them a prompt recovery after overcoming the stress period.

## 4. Materials and Methods

### 4.1. Plant Material and Growing Conditions

Cucumber (*Cucumis sativus* L. cv. Ashley) seeds were sterilized and placed on top of a filter paper which was moistened with a solution of CaSO_4_ 1 mM; then, the paper was rolled out and allowed to germinate in a growth chamber (Dycometal-type CCK) under controlled conditions (photoperiod 16/8 h (day/night), 25/20 °C, 40/60% relative humidity, and a photosynthetic photon flux density at the leaf of 1000 µmol·m^−2^∙s^−1^), with a CaSO_4_ 1 mM solution at the base of the paper. After one week of germination, uniform seeds were selected and transferred to 2 L plastic buckets, with nutrient solutions uninterruptedly aerated. The composition of the nutrient solution was: macronutrients (mM) Ca(NO_3_)_2_ (1.0), KNO_3_ (0.9), MgSO_4_ (0.1) and KH_2_PO_4_ (0.1); micronutrients (µM) NaCl (35), H_3_BO_3_ (10), Na_2_MoO_4_ (0.05), Na_2_-EDTA (Ethylenediamine tetraacetic acid) (115.5), MnSO_4_ (2.5), CuSO_4_ (1.0), ZnSO_4_ (1.0), CoSO_4_ (1.0), NiCl_2_ (1.0). Furthermore, 0.1 g/L of solid CaCO_3_ and 0.1 mM of HEPES (4-(2-hydroxyethyl)-1-piperazineethanesulfonic acid) was added to simulate conditions of calcareous soil; the pH was buffered to 7.5 and checked daily.

Iron chelate solution N,N′-bis(2-hydroxybenzyl)ethylenediamine-N,N′-diacetic acid (HBED) was prepared as described by [74], HBED was purchased from Strem Chemicals, ligand was dissolved in NaOH 1:4 molar ratio; subsequently, Fe(NO_3_)_3_ solution was added, pH was adjusted to 7.0, and the mixture was left to stand overnight, filtered, and made up to volume. Silicic acid (H_4_SiO_4_) was freshly prepared as described by [75] passing on Na_2_SiO_3_∙5 H_2_O (Sigma-Aldrich, Darmstadt, Germany), through a column containing a cation-exchange resin in its H+ form (Amberlite IR 120+, Sigma-Aldrich, Darmstadt, Germany).

Plants were grown in a full-strength nutrient solution continuously aerated for 1 week, and Si was applied as follows: A concentration of 1.5 mM of silicic acid was applied via foliar (+SiF) by spraying three doses (125 µL each) per leaf per week, dripping was not observed; the same concentration was applied through the root (+SiR) by adding silicic acid to the nutrient solution,; finally control plants without silicon (−Si) were also tested. Then, zinc deficiency (−Zn) conditions were induced in half of the plant material of each treatment, allowing them to grow during 9 days in zinc-free nutrient solution. Finally, Zn was re-added (−Zn(+Zn)) to the nutrient solution for 11 days more. Three replications of each treatment were performed; each replica had 3 plants. Two samplings were carried out: S1 at the end of the Zn deficiency period, and S2 at the end of the Zn re-fertilization period. In sampling 1: Zn sufficient plants [(+Zn): 16 days with Zn]; and Zn deficient plants [(−Zn): 7 days with Zn + 9 days without Zn] were collected; and in sampling 2: Zn sufficient plants [(+Zn(+Zn): 27 days with Zn] and Zn resupply plants [(−Zn(+Zn)): 7 days with Zn + 9 days without Zn + 11 days Zn re-fertilization] were obtained

### 4.2. Determinations

The degree of chlorosis of the leaves was quantified by a nondestructive method using the SPAD (Soil and Plant Analyzer Development) model 502 (Minolta Co., Osaka, Japan) digital chlorophyll meter.

Plant material was divided into root, stem, old leaves, and new leaves, then was washed with 0.1% Tween 80 (*v*/*v*) and 1% HCl (*v*/*v*), rinsed twice with distilled water, and fresh weight (FW) was determined. Subsequently, all the material was stored at −80 °C for stress analysis. Macronutrients and micronutrients concentration was quantified after microwave (CEM Corporation MARS 240/50; Matthews, NC, USA) digestion with HNO_3_ 65% and H_2_O_2_ 30% by Thermo Scientific Inductively Coupled Plasma Optical Emission Spectroscopy (ICP-OES, Thermo Fisher Scientific, Waltham, MA, USA). Phosphorus concentration was determined by Bray I method [76].

Analysis of reactive oxygen species (ROS) was performed using fresh plant material (0.2 g); this material was chopped into 2 mL 50 mM HEPES at pH 7. Then, the extract (50 µL) with 150 µL 50 mM HEPES and 4 µL 5 µM H_2_DCFDA (diacetate of 2′,7′-diclorodihydrofluorescein) (Molecular Probes, Invitrogen, Carlsbad, CA, USA) was mixed and incubated for 30 min at 37 °C in agitation (100 rpm). Thereafter, the extract was centrifuged at 1000 rpm for 10 min; the pellet was resuspended in 0.2 mL HEPES and incubated for 10 min more at 37 °C fluorescence intensity of DCF was measured using a fluorescence spectrophotometer (Cary Eclipse Fluorescence, Varian, Australia) at room temperature, with an excitation wavelength of 488 nm and emission filter between 500 and 600 nm ( excitation and emission slits width 5 nm). Relative ROS production was determined using the fluorescence intensity.

### 4.3. Statistical Analysis

Data were treated by one-way analysis of variance (ANOVA). Treatments were compared using Duncan’s test for *p* < 0.05. Statistical analysis was performed by the SPSS software for Windows (V.24.0; SPSS, Chicago, IL, USA).

## Figures and Tables

**Table 1:** Fresh weight distribution in plant tissues (%), and total fresh weight (FW, g) of cucumber plants with a root (+SiR) or shoot (+SiF) Si application and grown under different Zn nutritional statuses. Sampling 1: plants collected after a Zn deficiency period (−Zn) compared to the control plants grown with continuous Zn addition (+Zn), and sampling 2: plants collected after a Zn re-fertilization period (−Zn(+Zn)) and compared to their corresponding control plants (+Zn (+Zn)).

	Fresh Weight Distribution in Plant Tissues (%)				Total FW (g)
	Root	Stem	Old Leaves	New Leaves	
	**Sampling 1**				
**−Si+Zn**	47.40 ^b^	9.52 ^ab^	31.23 ^a^	11.85 ^ab^	11.56 ^a^
**+SiR+Zn**	50.94 ^b^	8.32 ^b^	33.58 ^a^	7.17 ^b^	9.62 ^a^
**+SiF+Zn**	55.26 ^a^	7.33 ^ab^	25.44 ^a^	11.97 ^a^	15.96 ^a^
**−Si−Zn**	45.62 ^c^	15.67 ^c^	11.06 ^b^	27.65 ^b^	2.17 ^b^
**+SiR−Zn**	43.70 ^c^	14.96 ^c^	4.72 ^b^	36.61 ^b^	2.54 ^b^
**+SiF−Zn**	38.24 ^c^	15.69 ^c^	12.25 ^b^	33.82 ^b^	2.04 ^b^
	**Sampling 2**				
**−Si+Zn(+Zn)**	35.62 ^c^	25.12 ^b^	14.81 ^b^	24.46 ^c^	31.81 ^c^
**+SiR+Zn(+Zn)**	51.45 ^b^	19.51 ^a^	12.01 ^a^	17.04 ^d^	72.95 ^b^
**+SiF+Zn(+Zn)**	57.48 ^a^	15.25 ^a^	11.28 ^a^	15.98 ^d^	96.1 ^a^
**−Si−Zn(+Zn)**	37.15 ^c^	22.55 ^c^	7.95 ^c^	32.35 ^b^	5.41 ^d^
**+SiR−Zn(+Zn)**	41.90 ^c^	16.50 ^c^	7.83 ^c^	33.76 ^b^	13.15 ^d^
**+SiF−Zn(+Zn)**	34.74 ^c^	18.36 ^c^	4.49 ^c^	42.40 ^a^	7.57 ^d^

The data are the mean ± SE (*n* = 3). Different letters in the same column for each sampling indicate significant differences according to Duncan’s test (*p* < 0.05).

**Table 2:** Effect of Si addition to the root (+SiR) or to the shoot (+SiF) of cucumber plants on reactive oxygen species increment (∆ROS (FU)) in the new leaves of plants harvested after a Zn deficiency period (sampling 1), followed by a Zn re-fertilization period (sampling 2), compared to their corresponding control plants, with a continuous Zn supply, and without Si (−Si).

	∆ROS (FU)
	**Zn Sufficiency** **(Sampling 2-Sampling 1)**
**−Si**	−182.47 ± 44.06 ^e^
**+SiR**	−223.23 ± 39.52 ^e^
**+SiF**	−220.93 ± 26.77 ^e^
	**Zn Resupply** **(Sampling 2)—Zn Deficiency (Sampling 1)**
**−Si**	202.68 ± 13.42 ^a^
**+SiR**	114.54 ± 21.77 ^b^
**+SiF**	−43.33 ± 8.45 ^c^

The data are the mean ± SE (*n* = 3). Different letters in the same column for sufficiency and resupply treatments, respectively, indicate significant differences, according to Duncan’s test (*p* < 0.05).

**Table 3:** Zinc (Zn) and phosphorus (P) distribution in plant organs (%) and Zn (µg g^−1^ DW) and P concentration (mg g^−1^ DW) in the whole plant under different Zn and Si treatments (no Si (−Si), root (+SiR), and foliar (+SiF) application): (a) After a Zn deficiency period (−Zn) compared to the control plants with continuous Zn addition (+Zn) (sampling 1) and (b) After a Zn re-fertilization period (−Zn(+Zn)), compared to their corresponding control plants (+Zn(+Zn)) (sampling 2).

	Zn Distribution in Plant Tissues (%)				P Distribution in Plant Tissues (%)			
	Root	Old Leaves	New Leaves	Total (µg/g)	Root	Old Leaves	New Leaves	Total (mg/g)
**Sampling 1**								
**−Si+Zn**	51.59 ^a^	21.28 ^n.s^	27.14 ^bc^	117.78 ^a^	33.49 ^cd^	20.36 ^c^	46.14 ^b^	4.18 ^b^
**+SiR+Zn**	45.97 ^bc^	18.13	35.90 ^abc^	94.48 ^ab^	32.381 ^b^	48.56 ^ab^	19.05 ^c^	6.75 ^ab^
**+SiF+Zn**	44.40 ^ab^	15.02	40.58 ^a^	118.57 ^a^	25.53 ^bcd^	31.99 ^bc^	42.46 ^c^	5.61 ^ab^
**−Si−Zn**	40.49 ^c^	29.50	30.00 ^c^	78.57 ^b^	34.60 ^d^	51.40 ^a^	13.99 ^c^	5.89 ^ab^
**+SiR−Zn**	42.65 ^c^	24.79	32.56 ^c^	80.37 ^b^	36.43 ^a^	47.75 ^abc^	15.82 ^c^	6.18 ^ab^
**+SiF−Zn**	40.06 ^bc^	20.67	39.28 ^ab^	110.75 ^a^	26.09 ^bc^	21.10 ^bc^	52.80 ^a^	9.45 ^a^
**Sampling 2**								
**−Si+Zn(+Zn)**	26.02 ^b^	22.30 ^ab^	51.68 ^a^	145.64 ^b^	14.36 ^c^	64.07 ^c^	21.55 ^b^	1.96 ^b^
**+SiR+Zn(+Zn)**	47.30 ^a^	15.43 ^b^	37.27 ^a^	170.64 ^ab^	59.82 ^ab^	15.87 ^c^	24.30 ^b^	7.91 ^a^
**+SiF+Zn(+Zn)**	37.33 ^a^	23.51 ^a^	39.16 ^a^	183.89 ^a^	23.27 ^c^	50.79 ^bc^	25.93 ^b^	3.57 ^b^
**−Si−Zn(+Zn)**	33.45 ^c^	25.77 ^b^	40.77 ^b^	93.20 ^c^	36.03 ^bc^	48.68 ^a^	15.27 ^b^	7.71 ^a^
**+SiR−Zn(+Zn)**	46.78 ^a^	15.73 ^b^	37.49 ^a^	164.31 ^ab^	41.42 ^a^	43.99 ^ab^	14.57 ^b^	6.70 ^a^
**+SiF−Zn(+Zn)**	34.80 ^b^	18.01 ^b^	47.19 ^a^	128.68 ^bc^	22.46 ^c^	29.96 ^bc^	47.57 ^a^	8.00 ^a^

The data are the mean ± SE (*n* = 3). Different letters in the same column for each sampling indicate significant differences according to Duncan’s test (*p* < 0.05).

**Table 4:** Copper (Cu) and boron (B) distribution in plant organs (%), and Cu and B concentration (µg g^−1^ DW) in the whole plant under different Zn and Si treatments (no Si (−Si), root (+SiR), and foliar (+SiF) application: (a) After a Zn deficiency period (−Zn) compared to the control plants with continuous Zn addition (+Zn) (sampling 1) and (b) After a Zn re-fertilization period (−Zn(+Zn)), compared to their corresponding control plants (+Zn(+Zn)) (sampling 2).

	Cu Distribution in Plant Tissues (%)				B Distribution in Plant Tissues (%)			
	Root	Old Leaves	New Leaves	Total (µg/g)	Root	Old Leaves	New Leaves	Total (µg/g)
**Sampling 1**								
**−Si+Zn**	29.12 ^bc^	27.42 ^a^	43.46 ^b^	84.31 ^c^	17.40 ^b^	68.22 ^a^	14.39 ^c^	49.51 ^ab^
**+SiR+Zn**	28.90 ^bc^	11.83 ^c^	59.26 ^a^	152.56 ^b^	32.64 ^a^	64.50 ^a^	2.86 ^d^	55.47 ^a^
**+SiF+Zn**	26.79 ^c^	14.91 ^c^	58.30 ^a^	77.85 ^c^	15.71 ^b^	62.41 ^a^	21.88 ^bc^	58.23 ^a^
**−Si−Zn**	35.16 ^b^	25.05 ^a^	39.79 ^c^	259.78 ^a^	10.82 ^b^	52.17 ^b^	37.01 ^a^	44.55 ^b^
**+SiR−Zn**	39.73 ^b^	12.93 ^c^	47.34 ^b^	168.67 ^b^	12.73 ^b^	51.61 ^b^	35.67 ^a^	43.72 ^b^
**+SiF−Zn**	59.50 ^a^	20.12 ^b^	20.38 ^d^	274.10 ^a^	12.77 ^b^	48.56 ^b^	38.66 ^a^	56.11 ^a^
**Sampling 2**								
**−Si+Zn(+Zn)**	33.01 ^d^	22.13 ^ab^	44.86 ^a^	81.78 ^c^	32.64 ^b^	46.31 ^b^	21.05 ^b^	70.29 ^b^
**+SiR+Zn(+Zn)**	48.91 ^c^	24.75 ^a^	26.34 ^c^	76.02 ^c^	12.77 ^c^	68.20 ^a^	19.03 ^b^	94.34 ^a^
**+SiF+Zn(+Zn)**	49.79 ^c^	22.79 ^ab^	27.43 ^c^	62.06 ^d^	28.79 ^b^	37.43 ^c^	33.78 ^a^	103.19 ^a^
**−Si−Zn(+Zn)**	60.67 ^b^	7.28 ^c^	32.05 ^b^	170.52 ^a^	26.84 ^c^	70.03 ^a^	3.13 ^c^	44.73 ^c^
**+SiR−Zn(+Zn)**	56.80 ^b^	18.51 ^b^	24.70 ^c^	86.43 ^c^	26.85 ^c^	36.95 ^c^	36.20 ^a^	57.93 ^c^
**+SiF−Zn(+Zn)**	71.57 ^a^	27.23 ^a^	1.20 ^d^	116.25 ^b^	38.78 ^a^	40.25 ^bc^	20.97 ^b^	108.87 ^a^

The data are the mean ± SE (*n* = 3). Different letters in the same column for each sampling indicate significant differences according to Duncan’s test (*p* < 0.05).

## Data Availability

The data presented in this study are available in the article.
